# Cocaine and Caffeine Effects on the Conditioned Place Preference Test: Concomitant Changes on Early Genes within the Mouse Prefrontal Cortex and Nucleus Accumbens

**DOI:** 10.3389/fnbeh.2017.00200

**Published:** 2017-10-18

**Authors:** Javier A. Muñiz, José P. Prieto, Betina González, Máximo H. Sosa, Jean L. Cadet, Cecilia Scorza, Francisco J. Urbano, Verónica Bisagno

**Affiliations:** ^1^Facultad de Farmacia y Bioquímica, Universidad de Buenos Aires, Buenos Aires, Argentina; ^2^Instituto de Investigaciones Farmacológicas, Universidad de Buenos Aires, Consejo Nacional de Investigaciones Científicas y Técnicas (CONICET), Buenos Aires, Argentina; ^3^Departamento de Neurofarmacología Experimental, Instituto de Investigaciones Biológicas Clemente Estable, Montevideo, Uruguay; ^4^National Institute on Drug Abuse (NIDA), Intramural Program, Molecular Neuropsychiatry Research Branch, Baltimore, MD, United States; ^5^Laboratorio de Fisiología y Biología Molecular, Instituto de Fisiología, Biología Molecular y Neurociencias, Universidad de Buenos Aires, Consejo Nacional de Investigaciones Científicas y Técnicas (CONICET), Buenos Aires, Argentina

**Keywords:** caffeine, cocaine, immediate-early genes, nucleus accumbens, prefrontal cortex, learning

## Abstract

Caffeine is the world's most popular psychostimulant and is frequently used as an active adulterant in many illicit drugs including cocaine. Previous studies have shown that caffeine can potentiate the stimulant effects of cocaine and cocaine-induced drug seeking behavior. However, little is known about the effects of this drug combination on reward-related learning, a key process in the maintenance of addiction and vulnerability to relapse. The goal of the present study was thus to determine caffeine and cocaine combined effects on the Conditioned Place Preference (CPP) test and to determine potential differential mRNA expression in the Nucleus Accumbens (NAc) and medial prefrontal cortex (mPFC) of immediate-early genes (IEGs) as well as dopamine and adenosine receptor subunits. Mice were treated with caffeine (5 mg/kg, CAF), cocaine (10 mg/kg, COC), or their combination (caffeine 5 mg/kg + cocaine 10 mg/kg, CAF-COC) and trained in the CPP test or treated with repeated injections inside the home cage. NAc and mPFC tissues were dissected immediately after the CPP test, after a single conditioning session or following psychostimulant injection in the home cage for mRNA expression analysis. CAF-COC induced a marked change of preference to the drug conditioned side of the CPP and a significant increase in locomotion compared to COC. Gene expression analysis after CPP test revealed specific up-regulation in the CAF-COC group of *Drd1a, cFos*, and *FosB* in the NAc, and *cFos, Egr1*, and *Npas4* in the mPFC. Importantly, none of these changes were observed when animals received same treatments in their home cage. With a single conditioning session, we found similar effects in both CAF and CAF-COC groups: increased *Drd1a* and decreased *cFos* in the NAc, and increased expression of *Drd1a* and *Drd2*, in the mPFC. Interestingly, we found that *cFos* and *Npas4* gene expression were increased only in the mPFC of the CAF-COC. Our study provides evidence that caffeine acting as an adulterant could potentiate reward-associated memories elicited by cocaine. This is associated with specific changes in IEGs expression that were observed almost exclusively in mice that received the combination of both psychostimulants in the context of CPP memory encoding and retrieval. Our results highlight the potential relevance of caffeine in the maintenance of cocaine addiction which might be mediated by modifying neural plasticity mechanisms that strengthen learning of the association between drug and environment.

## Introduction

Addictions are brain disorders that affect neural pathways involved in reward, motivation, and memory (Volkow et al., 2012). Recent neurobehavioral studies have shown that drug users show a number of cognitive deficits that may be secondary to drug-induced changes in brain structure and function (Cadet and Bisagno, 2016). Learned associations between the rewarding effects of a drug, and contextual stimuli present at the time of drug use, are a central component of acquiring and maintaining drug seeking behavior, and are capable of inducing relapse (Volkow et al., 2012). Understanding the mechanisms that underlie these unwanted memories should provide insight into effective therapies aimed at reducing context-induced relapse.

Cocaine is a highly addictive psychostimulant and is rarely obtained on the illegal market in its pure form (Cole et al., 2011). Forensic analyses of seized illicit cocaine samples usually report variable quantities of caffeine (López-Hill et al., 2011; Sena et al., 2017). Caffeine deserves a special consideration on its own. Energy drinks marketed as dietary supplements or beverages usually contain caffeine as their active ingredient (Sena et al., 2017). Such supplements are taken to enhance cognitive performance but might also influence mood and sleep (Childs, 2014). In clinical settings, caffeine intake is positively correlated with substance-use disorders (Kendler et al., 2006) and has been shown to increase illicit drug use (Miller, 2008).

In preclinical studies, we have previously demonstrated that caffeine can potentiate cocaine-mediated motor (Prieto et al., 2015; Muñiz et al., 2016) and motivational effects (Prieto et al., 2016). Also, caffeine potentiates striatal reactive astrocytosis induced by cocaine (Muñiz et al., 2016) or MDMA (Khairnar et al., 2010) and cocaine-mediated testicular toxicity (González et al., 2015).

Expression of immediate-early genes (IEGs) has been shown to be induced by activity-dependent synaptic plasticity or behavioral training and is thought to play an important role in memory storage (Cruz et al., 2015). IEGs encode regulatory transcription factors and a variety of proteins that act on diverse cellular processes (Cruz et al., 2013). Several IEGs have been of particular interest due to their association with synaptic plasticity and learning. These include *Egr1* (early growth response gene 1) (Veyrac et al., 2014) or *Npas4* (neuronal Per-Arnt-Sim domain protein 4) (Spiegel et al., 2014).

Findings reviewed above suggest that distinct neuroadaptations can emerge in the central nervous system (CNS) with the combination of caffeine and cocaine. At the basis of such neuroadaptations lie changes in the expression of IEGs. However, information is scarce regarding potential additive effects of these two psychostimulants on transcriptional regulators controlling genomic responses of activated neurons within the mesolimbic circuit. Therefore, in the present study we investigated changes in expression of IEGs (*cFos, FosB, cJun, Egr1, Npas4*) as well as Dopamine and Adenosine receptor subunits mRNA changes in the Nucleus Accumbens (NAc) and medial Prefrontal Cortex (mPFC), as a consequence of combined administration of caffeine and cocaine. We compared molecular changes induced by associative learning with both psychostimulants, using the conditioned place preference (CPP) test, with those obtained after repeated psychostimulant injections given in the home cage.

## Materials and methods

### Animals

Male C57BL/6 mice (10–12 weeks old) from the School of Exact and Natural Sciences of the University of Buenos Aires (UBA) were housed in a light- and temperature-controlled room. Mice had free access to food and water. We followed “Guidelines for the Care and Use of Mammals in Neuroscience and Behavioral Research” (National Research Council (US) Committee on Guidelines for the Use of Animals in Neuroscience and Behavioral Research, 2003) and approved by IACUC Committee of the Faculty of Pharmacy and Biochemistry, Universidad de Buenos Aires (Protocol Number: EXP_UBA N° 40944/20151). Mice were euthanized by cervical dislocation performed by an individual proficient in this technique.

### Pharmacological and physiological procedures

**Figure 1:**
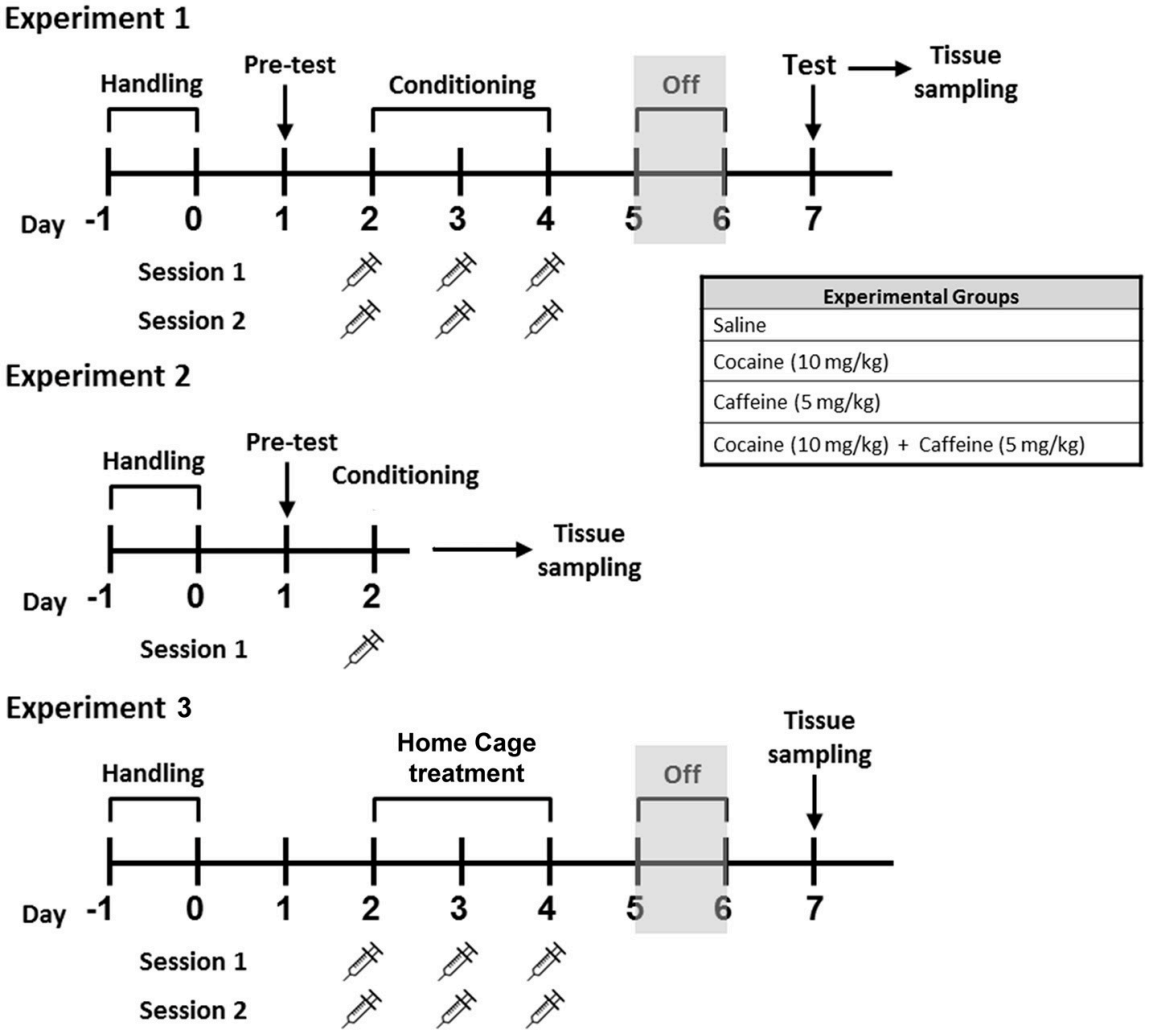
Schematic representation of the experimental treatments for Experiments #1, #2, and #3 Male C57BL/6 mice were subjected to CPP learning with caffeine 5 mg/kg (CAF), cocaine 10 mg/kg (COC), and their combination (5 mg/kg caffeine + 10 mg/kg cocaine, CAF-COC). Tissue samples for RT-PCR were taken immediately after the first conditioning CPP session, (Experiment #2), or immediately after CPP test session, (Experiment #1). For Experiment #3, brain tissue was obtained 48 h after last injection.

Cocaine hydrochloride and caffeine were purchased from Sigma-Aldrich, St. Louis, MO. Animals were assigned to four different groups: COC (Cocaine-only, 10 mg/kg), CAF (Caffeine-only, 5 mg/kg), CAF-COC (Combined, 10 mg/kg + Caffeine 5 mg/kg), both dissolved in sterile saline and co-administered in a single injection) or Control (sterile saline), see Figure 1.

Brain tissue, mPFC and NAc, were obtained immediately after CPP test ended (Experiment #1), or just after first conditioning session of the CPP test (Experiment #2). For Experiment #3, animals were injected with the same number of injections (saline and/or drug) used for CPP experiments but injections were made inside their home cage, and animals were sacrificed 48 h after last injection (following the same time schedule used for CPP, see Figure 1).

### Behavioral analysis

#### Conditioned place preference

**Figure 2:**
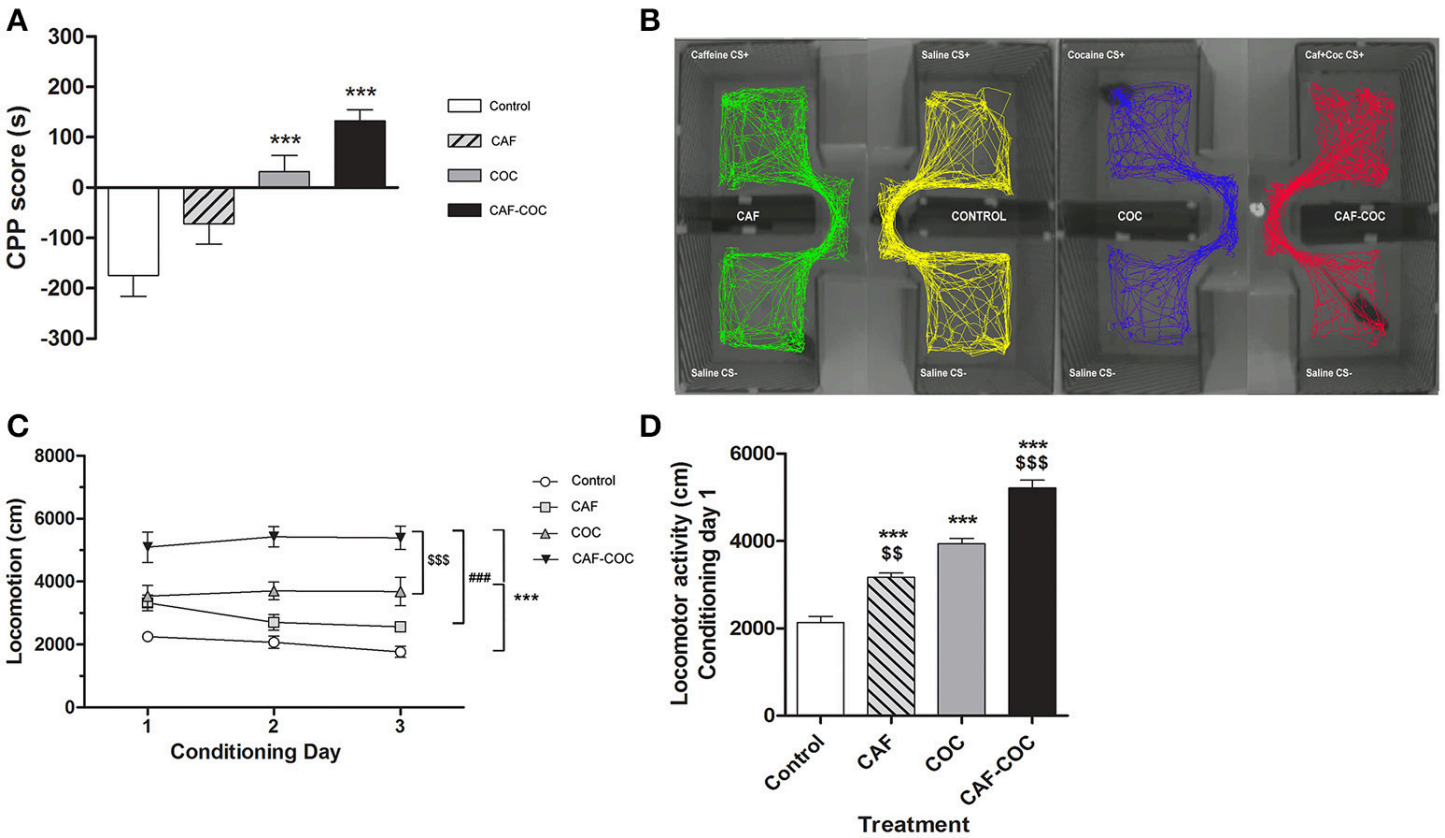
Behavioral changes induced by caffeine, cocaine or the combination of both psychostimulants. **(A)** CPP score calculated as follows: CPP score (s) = time spent in the CS + chamber – time spent in the CS − chamber. Values indicate mean ± SEM. One-way ANOVA–Tukey: ^***^p < 0.001 different from Control. **(B)** Representative track plots (screen-captured from Ethovision file) of mice during CPP test. **(C)** Psychostimulant treatments induced locomotor activity during the conditioning phase in the CS+ chamber. Two-way ANOVA with repeated measures followed by Bonferroni test, CAF-COC ^***^p < 0.001 different from Control, ^###^*p* < 0.001 different from CAF and ^$$$^*p* < 0.001 different from COC. **(D)** Locomotor activity measured on the first conditioning day session in the CS+ chamber. One-way ANOVA–Tukey. ^***^p < 0.001 different from Control; ^$$^*p* < 0.01, ^$$$^*p* < 0.001 different from COC.

A detailed plan of the training schedule is shown in Figure 1. CPP test was done in a custom-made plastic apparatus consisting of two equal size chambers (25 × 20 × 20 cm) connected by a smaller center chamber (25 × 7.5 × 18 cm) (see Figure 2B). Between the chambers were guillotine doors that were closed on the conditioning days and opened on the pretest and testing days. Both conditioning compartments had different visual, tactile and olfactory cues following a procedure previously described by Aguilar-Valles et al. (2014). First, animals were handled twice a day for 2 days before pretest trial. On the pretest session, animals were placed in the center chamber and allowed to explore freely for 15 min in order to establish the baseline preferences. Preference bias for any of the two conditioning compartments was declared based on the time spent in each compartment during the pretest session. Drug injections were paired with the initially non-preferred side (CS+ chamber), and saline was given in the preferred side (CS− chamber). On the following day, CPP conditioning started consisting in three consecutive training days with two daily training sessions (30 min). Injections were given just prior to placing the animal in the CS+ chamber and saline prior to the CS− chamber (chambers and time of day were counterbalanced between and within groups). Control animals received saline in both CS+ and CS− compartments. Forty-eight hours after the final training session, testing (15 min) consisted of giving mice free access to the apparatus in a drug-free state. CPP score was expressed as the time spent in the CS+ minus CS− compartments.

Experiment #2 was aimed to investigate changes during the initial encoding of the drug-context association. In this experiment, animals were exposed to the pretest session and then 24 h later received one CS+ pairing session in the morning. Brain tissue was collected immediately after the end of the first exposure to CS+.

Time spent in each chamber was recorded and analyzed using EthoVision XT 7.0 (Noldus) and confirmed by experimenter observation. Locomotor activity (total distance in cm) was recorded for all sessions while animals were inside CPP compartments.

### Real time PCR

Brain tissues were extracted after CPP test (Experiment #1), after CPP first conditioning (Experiment #2), or 48 h after last injection in the home cage (Experiment #3). Mouse brains were rapidly removed. mPFC and NAc were dissected, placed on dry ice, and then stored at −70°C in RNAlater (Qiagen) until further assays. Total RNA was isolated using TRIZOL reagent (Invitrogen) according to the manufacturer's protocol. Five hundred nanograms of RNA were treated with DNAseI (Invitrogen) and reverse-transcribed in a 20 μL reaction using M-MLV reverse transcriptase (Promega) and random hexamer (Biodynamics). For quantitative real-time PCR primers sets were designed for the specific amplification of murine *Drd1a, Drd2, Adora1, Adora2a, cFos, FosB, cJun, Egr1, Npas4*, and *Actb* as a housekeeping control gene (sequences listed in Table 1). Each sample was assayed in duplicate using 4 pmol of each primer, 1X SYBR Green Master Mix (Applied Biosystems), and 2–20 ng of cDNA in a total volume of 13 μL. Amplification was carried out in an ABI PRISM 7500 Sequence Detection System (Applied Biosystems).

**Table 1:** Primer sequences.

**Gene**	**Ac. Number**	**Primer forward**	**Primer reverse**
*Drd1a*	NM_010076	TTCTTCCTGGTATGGCTTGG	GCTTAGCCCTCACGTTCTTG
*Drd2*	NM_010077	TATGCCCTGGGTCGTCTATC	AGGACAGGACCCAGACAATG
*Adora1*	NM_001039510	TAGACAGTTCAGGTGGCCAG	AGTACATTTCCGGGCACAGA
*Adora2a*	NM_009630	CGCAGAGTTCCATCTTCAGC	ACGTCCTCAAACAGACAGGT
*cFos*	NM_010234	TCCCCAAACTTCGACCATGA	AGTTGGCACTAGAGACGGAC
*FosB*	NM_008036	ACAGATCGACTTCAG GCGGA	GTTTGTGGGCCACCAGGAC
*cJun*	NM_010591	CATAGCCAGAACACGCTTCC	TTGAAGTTGCTGAGGTTGGC
*Egr1*	NM_007913	GATGGTGGAGACGAGTTAT	GATTGGTCATGCTCACG
*Npas4*	NM_153553	CATCTGGGCCACTCTATGGT	AGGGGTCTCTTCTCTCCAGT
*Actb*	NM_007393	TGTTACCAACTGGGACGACA	GGGGTGTTGAAGGTCTCAAA

### Statistical analysis

Several statistical analyses were performed with ANOVAs in order to detect and better characterize possible differences between the experimental conditions. Before performing ANOVAs, data sets of each of the different experimental variables were inspected for homogeneity of variances and normal distribution among the four experimental groups. Transformation of data was applied to bring data to normal distribution when required. Kruskal Wallis ANOVA on Ranks was performed when data did not comply with the assumptions of parametric tests. When one-way ANOVA was performed, Tukey was used as the post-hoc test (http://www.infostat.com.ar/). Infostat software by default applies Tukey-Kramer test for unbalanced designs when needed. Data are expressed as the mean ± SEM. Differences were considered significant if *p* < 0.05.

For statistical analysis of locomotor activity of Experiment #1, two-way ANOVA with repeated measures (treatment and day effects) followed by Bonferroni test was applied using the software IBM SPSS Statistics V20.0.0. Bonferroni correction allows controlling α error by adjusting the level of significance of each individual contrast according to the number of comparisons.

## Results

### Experiment #1: cocaine and caffeine-induced behavioral and molecular effects following the CPP test

#### 1a. CPP

All animals established a preference for one of the two conditioning compartments of the CPP box at the pretest day, i.e., before place conditioning was initiated. Animals assigned to the drug group were conditioned with drugs to the non-preferred compartment (CS+ chamber). As shown in Figure 2A, mice in the COC group developed a positive CPP for the cocaine associated compartment at the CPP test day [*F*_(3, 38)_ = 14.48 *p* < 0.001, *N* = 9–11, Tukey: *p* < 0.001 different from Control]. CAF-COC also induced a positive CPP demonstrated by a marked preference for the initially non-preferred side following association with drug administration (Tukey: *p* < 0.001 different from Control). For caffeine treated mice, no statistical differences were found compared to the Control group (Tukey: *p* > 0.05). Figure 2B shows representative track plots (screen-captured from EthoVision file) of mice during CPP test. Previous work has indicated that when combined, low-doses of cocaine and caffeine enhance approach behavior to cues paired with their administration in rats, suggesting that reinforcement induced by these drugs may be additive (Bedingfield et al., 1998). Our results also show a tendency of caffeine to increase CPP score values (compared with cocaine values) but the doses tested in this study did not show a clear additive effect (Figure 2A).

In addition, locomotor activity was measured during the conditioning phase in the CS+ chamber (Figure 2C). Two-way ANOVA with repeated measures showed significant main treatment effect [*F*_(3, 40)_ = 29.18, *p* < 0.001, Bonferroni test for multiple comparisons: CAF-COC *p* < 0.001 different from Control; *p* < 0.001 different from CAF; *p* < 0.001 different from COC; COC *p* < 0.001 different from Control]. However, no significant effect of day [*F*_(2, 80)_ = 0.86, *p* > 0.05, *N* = 10–12], or day by treatment interaction [*F*_(6, 80)_ = 1.62, *p* > 0.05] were observed. As depicted in Figure 2D, mice receiving the combination of CAF-COC on day one of conditioning were significantly more active than animals administered with any other treatment.

#### 1.b. gene expression changes induced by caffeine and cocaine-associated memory retrieval in the CPP test

Tissue samples from the mPFC and NAc were obtained for gene expression analysis immediately following CPP testing, as shown in Figure 1. We measured mRNA expression of Dopamine and Adenosine receptor subunits and IEGs that are sensitive to drug exposure and associated with neuronal activation, plasticity and memory.

##### mPFC

**Figure 3:**
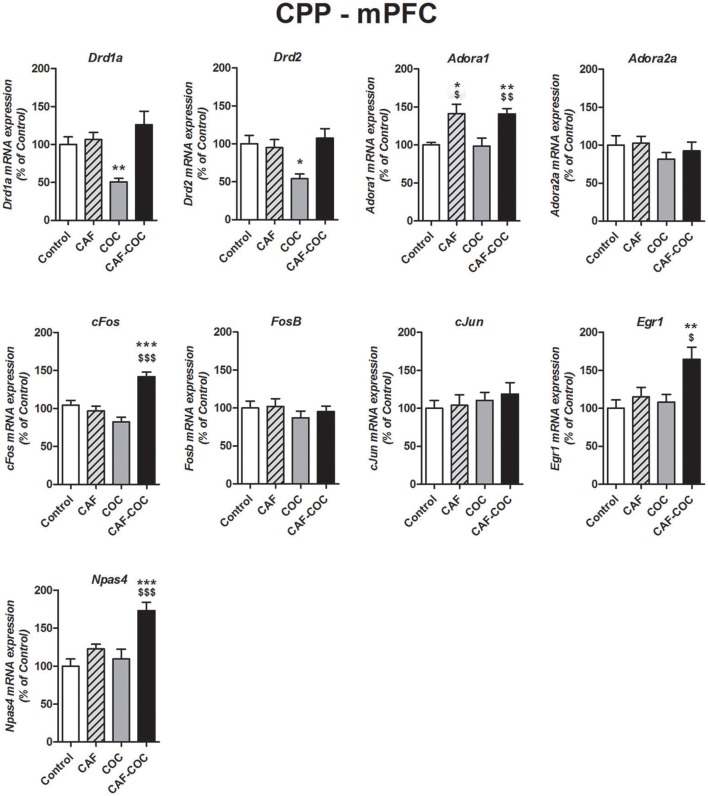
Gene expression changes induced in the mPFC by caffeine and cocaine-associated memory retrieval in the CPP. Dopamine receptor subunits *Drd1a* and *Drd2*; adenosine receptor subunits *Adora1* and *Adora2*a; IEGs *FosB, cFos, cJun, Egr1, Npas4*. The values indicate mean ± SEM. One-way ANOVA–Tukey: ^*^p < 0.05, ^**^p < 0.01, ^***^p < 0.001 different from Control; ^$^*p* < 0.05, ^$$^*p* < 0.01, ^$$$^*p* < 0.001 different from COC.

Figure 3 shows gene expression effects following CPP with caffeine, cocaine, and their combination on Dopamine and Adenosine receptor subunit and IEGs mRNA expression in the mPFC. One-way ANOVA-Tukey indicated that cocaine given alone decreased Dopamine receptor subunits, *Drd1a* [*F*_(3, 19)_ = 7.88, *p* < 0.01, *N* = 5, Tukey: *p* < 0.01 different from Control] and *Drd2* [*F*_(3, 21)_ = 5.73, *p* < 0.01, *N* = 5–6, Tukey: *p* < 0.05 different from Control], in the mPFC. For the Adenosine receptor subunits, both groups that were trained in the CPP with caffeine had increased expression of *Adora1* [*F*_(3, 21)_ = 6.97, *p* < 0.01, *N* = 5–6, Tukey: CAF *p* < 0.05, CAF-COC *p* < 0.01 different from Control; CAF *p* < 0.05, CAF-COC *p* < 0.01 different from COC], whereas there were no changes in *Adora2a* gene expression [*F*_(3, 22)_ = 0.82, *p* > 0.05 *N* = 5–6]. Interestingly, three out of five IEGs including *cFos, Egr1*, and *Npas4* showed significant increases solely in the group that was trained with the combination of both psychostimulants {*cFos* [*F*_(3 22)_ = 18.41, *p* < 0.001, *N* = 5–6, Tukey: *p* < 0.001 different from Control; *p* < 0.001 different from COC]; *Egr1* [*F*_(3, 23)_ = 5.27, *p* < 0.01, *N* = 6, Tukey: *p* < 0.01 different from Control; *p* < 0.05 different from COC]; *Npas4* [*F*_(3, 23)_ = 10.57, *p* < 0.001 *N* = 6, Tukey: *p* < 0.001 different from Control; *p* < 0.001 different from COC]}. No significant differences were found for *cJun* [*F*_(3, 22)_ = 0.42, *p* > 0.05, *N* = 5–6] and *FosB* [*F*_(3, 22)_ = 0.64, *p* > 0.05, *N* = 5–6] expression in the mPFC.

##### NAc

**Figure 4:**
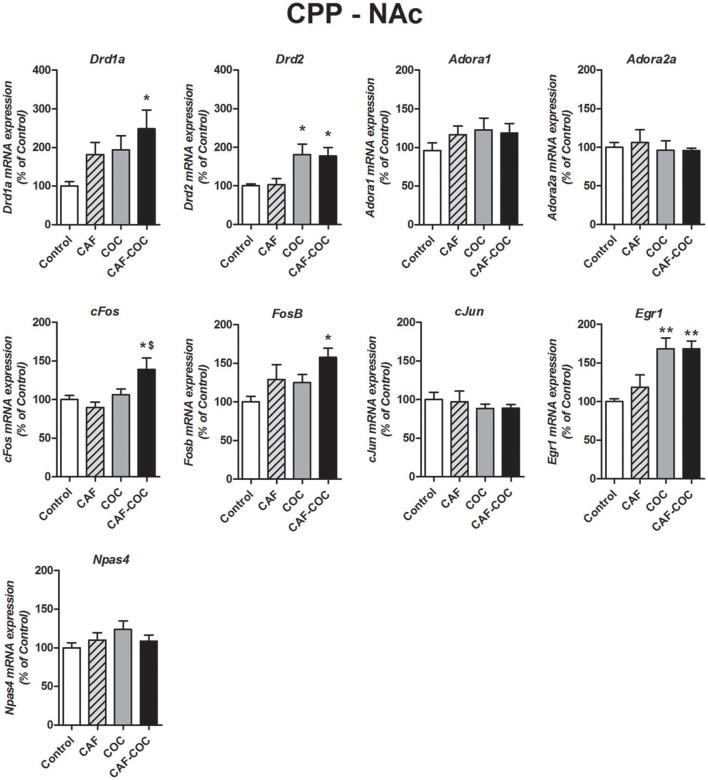
Gene expression changes induced in the NAc by caffeine and cocaine-associated memory retrieval in the CPP. Dopamine receptor subunits *Drd1a* and *Drd2*; adenosine receptor subunits *Adora1* and *Adora2a*; IEGs *FosB, cFos, cJun, Egr1, Npas4*. Values indicate mean ± SEM. One-way ANOVA–Tukey. Kruskal–Wallis One–way ANOVA on ranks was used for *Drd2* data. ^*^p < 0.05, ^**^p < 0.01 different from Control. ^$^*p* < 0.05, different from COC.

Figure 4 shows gene expression effects following CPP with caffeine, cocaine, and their combination on Dopamine and Adenosine receptor subunit and IEGs mRNA expression in the NAc. Caffeine or cocaine given alone did not modify Dopamine receptor subunits *Drd1a* but the combination of both drugs (CAF-COC) induced and increase in *Drd1a* expression [*F*_(3, 19)_ = 3.31, *p* < 0.05, *N* = 4–5, Tukey: *p* < 0.05 different from Control]. For *Drd2*, both COC and CAF-COC groups induced an increase in *Drd2* [Kruskal-Wallis H = 11.68, *p* < 0.01, *N* = 4–5, paired comparisons: *p* < 0.05 different from Control] suggesting a cocaine-mediated effect. We did not find any significant effects for Adenosine receptor subunits *Adora1* [*F*_(3, 20)_ = 0.87, *p* > 0.05, *N* = 5–6] and *Adora2a* [*F*_(3, 19)_ = 0.22, *p* > 0.05, *N* = 4–6] for any experimental group in the NAc.

*FosB* and *cFos* only increased in the group that received the combination of both psychostimulants [*FosB F*_(3, 20)_ = 5.272, *p* < 0.05, *N* = 5–6, Tukey: *p* < 0.05 different from Control; *p* < 0.05, different from COC. *cFos F*_(3, 20)_ = 18.41, *p* < 0.01, *N* = 5–6, Tukey: *p* < 0.05 different from Control]. Also, both COC and CAF-COC groups showed increased *Egr1* [*F*_(3, 19)_ = 5.27, *p* < 0.01, *N* = 4–6, Tukey: *p* < 0.01 different from Control]. CAF-COC treatment did not produce significant changes in *Npas4* and *cJun* expression [*Npas4 F*_(3, 20)_ = 1.17, *p* > 0.05, *N* = 5–6; *cJun F*_(3, 21)_ = 0.45, *p* > 0.05, *N* = 5–6].

### Experiment #2: cocaine and caffeine-induced behavioral and molecular effects following a single conditioning session of the CPP test

#### 2a. single conditioning session of the CPP test

Mice were handled for habituation, exposed to CPP pre-test and then exposed to CPP first conditioning session 24 h later, see Figure 1. We measured mRNA levels of the same genes previously described in the mPFC and NAc but in this experiment brain tissues were obtained immediately after first CPP conditioning session. The main goal of this new experiment was to compare gene expression profile from a single exposure to the context with the drug or saline to the one observed following a full CPP test.

Treatments with both psychostimulants induced locomotor activity on the first conditioning session in the CS+ chamber, see Figure 2D. One-way ANOVA-Tukey [*F*_(3, 26)_ = 87.20, *p* < 0.001, *N* = 6–7] indicated that all groups showed increased locomotion compared to Control (*p* < 0.001 different from Control). Also, CAF and CAF-COC groups were different from COC group (*p* < 0.01 and *p* < 0.001 respectively).

#### 2b. gene expression changes following a single conditioning session of the CPP test

##### mPFC

**Figure 5:**
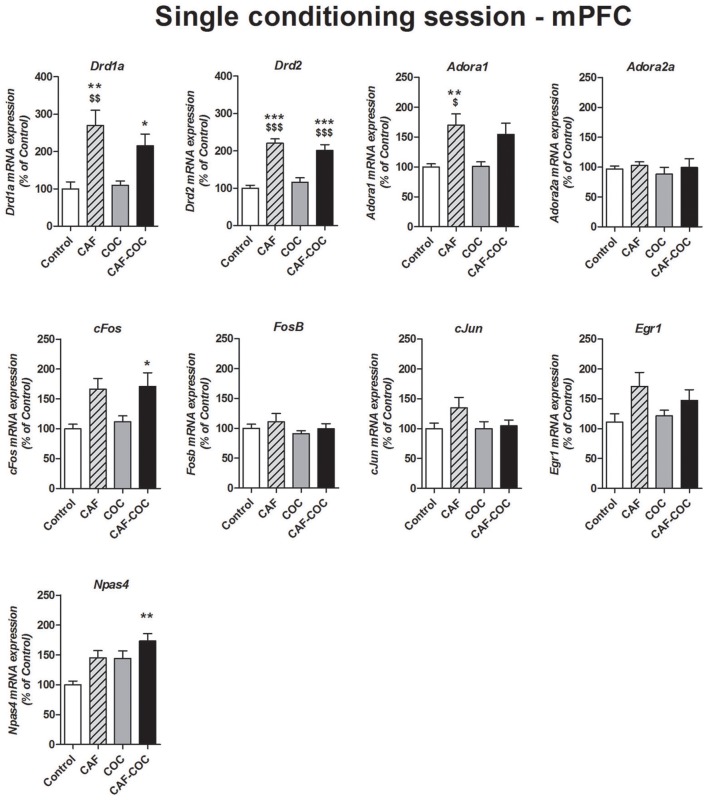
Gene expression changes in the mPFC following a single conditioning session of the CPP. Dopamine receptor subunits *Drd1a* and *Drd2*; adenosine receptor subunits *Adora1* and *Adora2a*; IEGs *FosB, cFos, cJun, Egr1, Npas4*. Values indicate mean ± SEM. One-way ANOVA–Tukey: ^*^p < 0.05, ^**^p < 0.01, ^***^p < 0.001 different from Control; ^$^*p* < 0.05, ^$$^*p* < 0.01, ^$$$^*p* < 0.001 different from COC.

Figure 5 shows gene expression changes following a single CPP encoding session with caffeine, cocaine, and their combination on Dopamine and Adenosine receptor subunits and IEGs mRNA expression in the mPFC. One-way ANOVA-Tukey indicated that groups that received caffeine, given alone or in combination with cocaine, showed significant increase in Dopamine receptor subunits *Drd1a* and *Drd2* in mPFC [*Drd1a F*_(3, 21)_ = 8.43, *p* < 0.01, *N* = 5–6, Tukey: CAF *p* < 0.05, CAF-COC *p* < 0.01 different from Control; CAF *p* < 0.01 different from COC; *Drd2 F*_(3, 21)_ = 23.09, *p* < 0.001, *N* = 5–6, Tukey: CAF and CAF-COC *p* < 0.001 different from Control; *p* < 0.001 different from COC]. For *Adora1* we found increased expression in the CAF group [*F*_(3, 21)_ = 6.75, *p* < 0.01, *N* = 5–6 Tukey: *p* < 0.01 different from Control; *p* < 0.05 different from COC]. No changes were observed in *Adora2a* gene expression in any groups [*F*_(3, 22)_ = 0.35, *p* > 0.05, *N* = 5–6]. Concerning IEGs expression, for *cFos* and *Npas4* we observed the same trend that the one observed following full CPP, meaning that only groups that received the combination of CAF-COC showed increased expression [*cFos F*_(3, 20)_ = 4.73, *p* < 0.05, *N* = 5–6, Tukey: *p* < 0.05 different from Control; *Npas4 F*_(3, 22)_ = 6.46, *p* < 0.01, *N* = 5–6, Tukey: *p* < 0.01 different from Control]. No significant differences were found for *cJun, Egr1* or *FosB* expression in the mPFC [*cJun F*_(3, 23)_ = 1.82, *p* > 0.05, *N* = 6; *Egr1 F*_(3, 23)_ = 2.55, *p* > 0.05, *N* = 6; *FosB F*_(3, 23)_ = 0.77, *p* > 0.05, *N* = 6].

##### NAc

**Figure 6:**
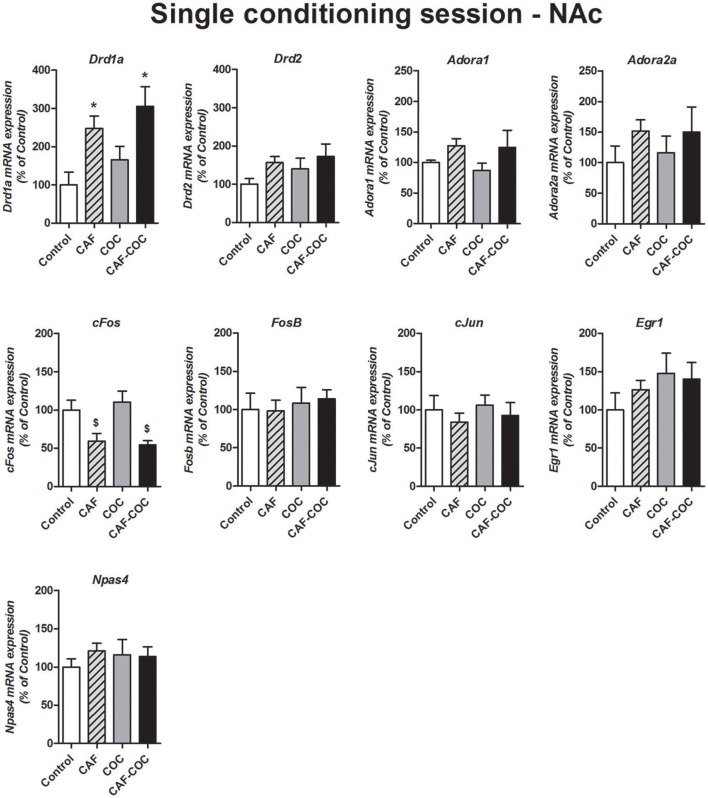
Gene expression changes in the NAc following a single conditioning session of the CPP. Dopamine receptor subunits *Drd1a* and *Drd2*; adenosine receptor subunits *Adora1* and *Adora2a*; IEGs *FosB, cFos, cJun, Egr1, Npas4*. Values indicate mean ± SEM. One-way ANOVA–Tukey: ^*^p < 0.05 different from Control, ^$^*p* < 0.05 different from COC.

Figure 6 shows gene expression changes following a single CPP encoding session with caffeine, cocaine, and their combination on Dopamine and Adenosine receptor subunits and IEGs mRNA expression in the NAc. Overall, we found fewer gene expression changes (compared to the profile obtained after CPP test). The only genes that showed changes were *Drd1a* and *cFos*. For *Drd1a*, we found that caffeine induced increases in *Drd1a*, given alone or in combination [*F*_(3, 20)_ = 5.68, *p* < 0.01, *N* = 4–6, Tukey: CAF and CAF-COC *p* < 0.05 different from Control]. For *cFos*, we also observed a caffeine-mediated effect but in this case, caffeine-treated groups showed a decrease in its expression compared to COC group [*F*_(3, 19)_ = 5.87, *p* < 0.01, *N* = 4–6, Tukey: *p* < 0.05 different from COC]. We did not observe any other significant change on other genes: *Adora1* [*F*_(3, 19)_ = 1.43, *p* > 0.05, *N* = 4–5]; *Adora2a* [*F*_(3, 20)_ = 0.65, *p* > 0.05, *N* = 4–6]; *Drd2* [*F*_(3, 18)_ = 1.11, *p* > 0.05, *N* = 4–5]; *FosB* [*F*_(3, 21)_ = 0.17, *p* > 0.05, *N* = 5–6]; *cJun* [*F*_(3, 20)_ = 0.46, *p* > 0.05, *N* = 4–6]; *Egr1* [*F*_(3, 21)_ = 0.93, *p* > 0.05, *N* = 5–6; *Npas4* [*F*_(3, 21)_ = 0.37, *p* > 0.05, *N* = 5–6].

### Experiment #3: gene expression changes induced by caffeine and cocaine repeated treatment in the home cage

In Experiment #3, mice received the same handling and the same doses and number of injections (one injection with saline plus one injection with drug or saline per day, for 3 consecutive days) as the groups that were trained and tested in the CPP (Experiment #1) but injections were given in the home cage, see Figure 1. Following the CPP protocol, animals were left undisturbed in their home cage for 2 days. Animals were sacrificed 24 h later (in a drug free state) and brain tissue collected for gene expression analysis. The objective of the Experiment #3 was to investigate whether changes observed following the CPP test (i.e., in Experiment #1) were dependent on context or linked to drug administration (i.e., a consequence of repeated drug injection).

#### mPFC

**Figure 7:**
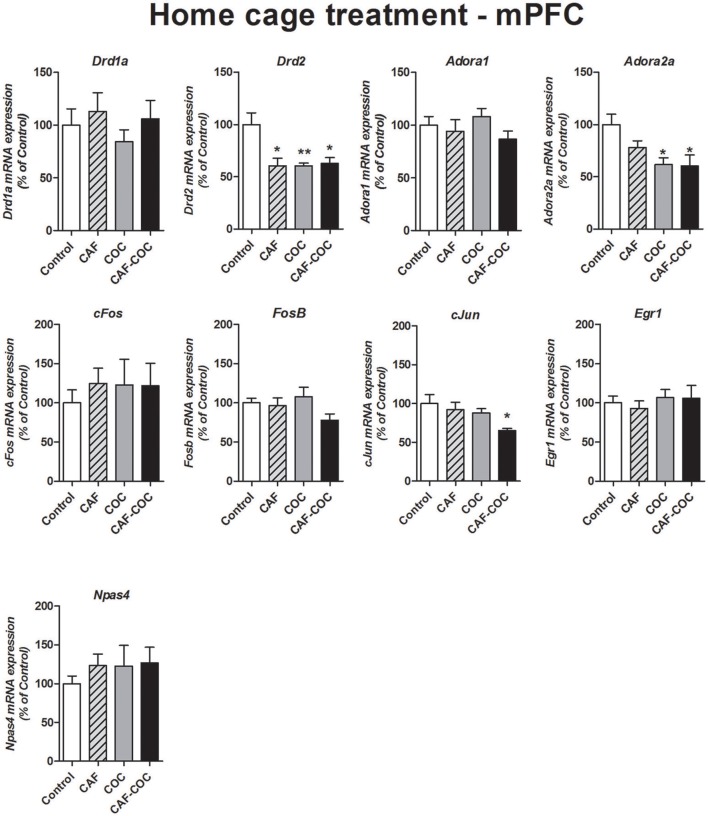
Gene expression changes in the mPFC induced by caffeine and cocaine repeated treatment in the home cage. Dopamine receptor subunits *Drd1a* and *Drd2*; adenosine receptor subunits *Adora1* and *Adora2a*; IEGs *FosB, cFos, cJun, Egr1, Npas4*. Values indicate mean ± SEM. One-way ANOVA–Tukey: ^*^p < 0.05, ^**^p < 0.01 different from Control.

Figure 7 shows changes in gene expression following a 3-day repeated treatment given in the home cage with caffeine, cocaine, and their combination in the mPFC. We measured Dopamine and Adenosine receptor subunits and IEGs mRNA expression from brain tissues obtained 48 h after the last injection, in a drug-free state. We have found decreased *Drd2* expression for all treatments [*F*_(3, 21)_ = 6.64, *p* < 0.01, *N* = 5–6, Tukey: *p* < 0.05 and *p* < 0.01 different from Control] and decreased *Adora2a* expression in cocaine treated groups [*F*_(3, 22)_ = 4.62, *p* < 0.05, *N* = 5–6, Tukey: *p* < 0.05 different from Control]. Also, the group injected with the combination of CAF-COC showed a significant decrease in *cJun* mRNA in the mPFC [*F*_(3, 23)_ = 3.28, *p* < 0.05, *N* = 6, Tukey: *p* < 0.05 different from Control]. No other significant changes were observed for *Adora1* [*F*_(3, 22)_ = 1.17, *p* > 0.05, *N* = 5–6], *Drd1a* [*F*_(3, 22)_ = 0.60, *p* > 0.05, *N* = 5–6], *FosB* [*F*_(3, 21)_ = 2.10, *p* > 0.05, *N* = 5–6], *cFos* [*F*_(3, 21)_ = 0.22, *p* > 0.05, *N* = 5–6], *Egr1* [*F*_(3, 20)_ = 0.29, *p* > 0.05, *N* = 5–6] and *Npas4* [*F*_(3, 21)_ = 0.48, *p* > 0.05, *N* = 5–6].

#### NAc

**Figure 8:**
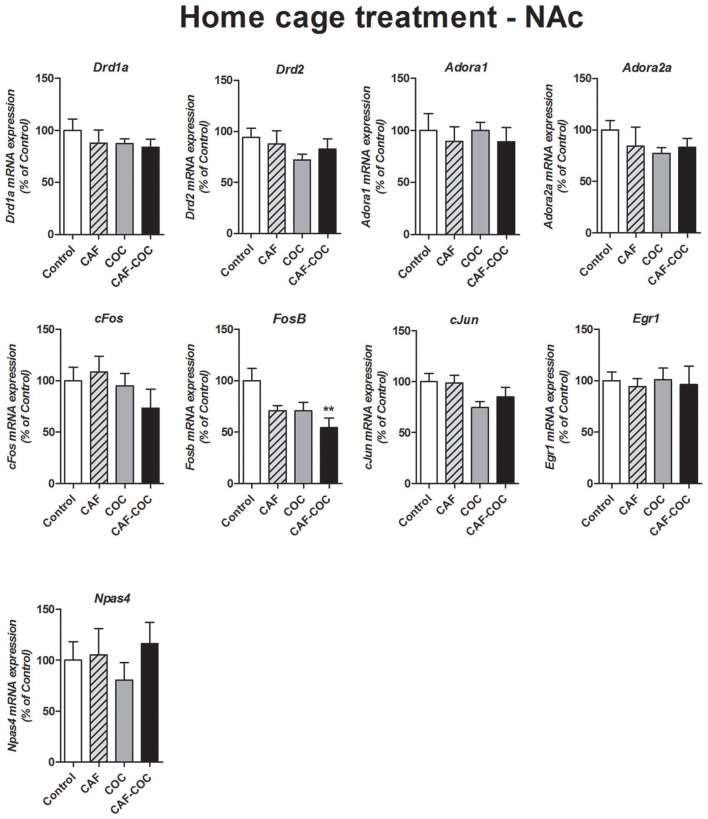
Gene expression changes in the NAc induced by caffeine and cocaine repeated treatment in the home cage. Dopamine receptor subunits *Drd1a* and *Drd2*; adenosine receptor subunits *Adora1* and *Adora2a*; IEGs *FosB, cFos, cJun, Egr1, Npas4*. Values indicate mean ± SEM. One-way ANOVA–Tukey: ^**^p < 0.01 different from Control.

Figure 8 shows gene expression changes following repeated treatment given in the home cage with caffeine, cocaine, and their combination, in the NAc. We measured Dopamine and Adenosine receptor subunits and IEGs mRNA expression from brain tissues obtained 48 h after the last injection, in a drug-free state. Overall, in Experiment #3 few changes were found on gene expression. No changes were found in many of the gene expression tested except for *FosB*. One-way ANOVA–Tukey indicated that the only gene demonstrating significant changes was *FosB*, a significant decrease was found [*F*_(3, 23)_ = 4.42, *p* < 0.05, *N* = 6 Tukey: *p* < 0.05 different from Control]. No other significant changes were observed for *Adora1* [*F*_(3, 23)_ = 0.21, *p* > 0.05, *N* = 6], *Adora2a* [*F*_(3, 20)_ = 0.78, *p* > 0.05, *N* = 5–6], *Drd1a* [*F*_(3, 19)_ = 0.55, *p* > 0.05, *N* = 5]; *Drd2* [*F*_(3, 20)_ = 0.79, *p* > 0.05, *N* = 5–6], *cFos* [*F*_(3, 22)_ = 1.01, *p* > 0.05, *N* = 5–6], *cJun* [*F*_(3, 22)_ = 2.23, *p* > 0.05, *N* = 5–6], *Egr1* [*F*_(3, 23)_ = 0.06, *p* > 0.05, *N* = 6] and *Npas4* [*F*_(3, 21)_ = 0.45, *p* > 0.05, *N* = 5–6].

Gene expression data for all experimental conditions are summarized in Table 2.

**Table 2:** Gene expression change for all experimental conditions: FC (first conditioning CPP session), CPP (CPP test session) or HC (injections in home cage).

**mPFC**		**FC**			**CPP**			**HC**		
		**Caf**	**Coc**	**Caf-Coc**	**Caf**	**Coc**	**Caf-Coc**	**Caf**	**Coc**	**Caf-Coc**
	*Drd1a*	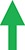	–	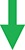	–	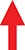	–	–	–	–
	*Drd2*	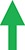	–	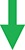	–	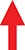	–	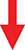	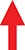	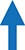
	*Adora1*	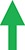	–	–	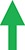	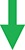	–	–	–	–
	*Adora2a*	–	–	–	–	–	–	–	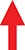	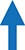
	*cFos*	–	–	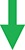	–	–	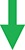	–	–	–
	*FosB*	–	–	–	–	–	–	–	–	–
	*cJun*	–	–	–	–	–	–	–	–	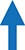
	*Egr1*	–	–	–	–	–	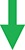	–	–	–
	*Npas4*	–	–	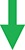	–	–	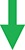	–	–	–
**NAc**	*Drd1a*	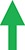	–	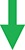	–	–	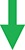	–	–	–
	*Drd2*	–	–	–	–	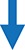	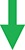	–	–	–
	*Adora1*	–	–	–	–	–	–	–	–	–
	*Adora2a*	–	–	–	–	–	–	–	–	–
	*cFos*	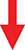	–	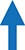	–	–	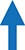	–	–	–
	*FosB*	–	–	–	–	–	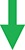	–	–	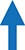
	*cJun*	–	–	–	–	–	–	–	–	–
	*Egr1*	–	–	–	–	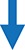	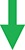	–	–	–
	*Npas4*	–	–	–	–	–	–	–	–	–

*Symbol order: first symbol (↑, increase; ↓, decrease or –, no change) relates to Caffeine (green), second symbol to Cocaine (blue) and third symbol to Caffeine-Cocaine (red) groups*.

## Discussion

We report three main findings of the present study. (1) Combined administration of caffeine and cocaine induced a marked change of preference to the drug conditioned side of the CPP and a significant increase in locomotion. (2) Molecular changes observed at the time of CPP memory retrieval revealed specific effects on IEGs mRNA that were present almost exclusively in mice that received the combination of both psychostimulants. (3) Neuroadaptations mediated by both psychostimulants implicated different changes in mPFC or NAc, and were dependent on context (CPP or home-cage administration). These findings are of interest as neuroimaging studies involving human subjects have associated functional changes in the mPFC (Volkow et al., 2005) and NAc (Risinger et al., 2005) with cocaine craving.

Psychostimulants are drugs that can act as cognitive enhancers and potentiate learning and memory (Bisagno et al., 2016). Positive effects of caffeine are well known and include enhanced wakefulness, increased concentration, and stimulated activity (Daly and Fredholm, 1998). It is also known that the drug administration environment is critical in determining which neuronal ensembles are activated during cocaine treatment (Mattson et al., 2008). Moreover, formation and storage of memory representations rely on activity-driven changes in synaptic plasticity of neural networks mediated by IEGs (Veyrac et al., 2014). We found distinct changes in a subset of IEGs (*cFos, FosB, cJun, Egr1*, and *Npas4*) induced by caffeine and cocaine administration in the mouse brain that may underlie neuroplasticity in the NAc and mPFC circuits. The potential implications for these differential changes in gene expression are discussed below.

### NAc

The NAc modulates the brain's natural reward system through changes in accumbal Dopamine released in response to rewarding stimuli that include motivation and incentivized learning (Cadet and Bisagno, 2016). CPP test trial responses depend on whether the animal remembers and/or retrieves the respective associations established during the drug-pairing sessions. In the present study we report that, while both groups that received cocaine showed positive CPP scores, only the group that received the combination of both psychostimulants during CPP training showed increased *cFo*s and *FosB* mRNA in the NAc. These changes appear to be context-induced since they did not occur in the groups treated with the same drug doses and schedule but in their home cage. In fact, we only found reduced *FosB* mRNA levels in the NAc of animals that received home cage treatment.

Drug-induced Dopamine can synergistically enhance *cFos* promoter activation in striatal neurons and those Fos-expressing neurons may act as a unit to form neuronal ensembles that encode conditioned drug behaviors (Cruz et al., 2015). ΔFosB, encoded by the FosB gene, is a member of the Fos family of IEGs induced in the NAc by the repeated administration of cocaine (Alibhai et al., 2007). It is important to note that the ability of ΔFosB to accumulate after a single exposure to cocaine is dependent on both the strength and duration of the stimulus (Kelz et al., 1999). This suggests that neuronal activity must reach a threshold for ΔFosB to accumulate in the NAc. This “all or nothing” theory was suggested for *cFos* since it is likely that only a small proportion of neurons experience enough strong activation so that calcium concentration surpasses the threshold for induction of *cFos* and other IEGs (Cruz et al., 2015). Our results suggest that the association of context with reward was not robust enough in mice receiving only one psychostimulant (at the doses used in the present study) to increase *cFos* or *FosB*. Accordingly, CPP training with the combination of caffeine plus cocaine is likely to produce a stronger stimulus that is sufficient to induce expression of these IEGs.

Expression of *Egr1* in the NAc was shown to be responsive to CPP training with cocaine (Fritz et al., 2011). Our results are also in agreement with a recent report by Li et al. (2016) who showed that *Egr1* and *FosB* in the NAc mediate memory of cocaine-place association. Moreover, these authors suggest that both D1 and NMDA receptors regulate these two IEGs during drug memory consolidation (Li et al., 2016). Interestingly, we also found that following CPP, only caffeine plus cocaine treated animals showed increased D1 receptor mRNA in the NAc.

Regarding caffeine-mediated effects, we found a decrease in *cFos* gene expression in the NAc of animals exposed to the first conditioning session that might be related to a decrease in NAc activation mediated by caffeine. It is important to note that the caffeine dose used in our study (5 mg/kg) is considered to be in the low range and other studies have reported that only high caffeine doses (i.e., 75 mg/kg) increase IEGs expression in the NAc (Bennett and Semba, 1998). In addition, significant increases in ΔFosB expression were observed in the NAc from animals exposed to caffeine mixed with alcohol, but not mice exposed to alcohol or caffeine (15 mg/kg) alone (Robins et al., 2016). These authors suggested that the combination of both drugs, i.e., caffeine and alcohol, increased Dopamine in the NAc to a higher degree than the stimulation produced by caffeine alone thereby increasing ΔFosB expression (Robins et al., 2016). We have also observed changes in the mRNAs for Adenosine and Dopamine receptors that appear to be sensitive to psychostimulants in striatal tissue (Ferré et al., 2010; Volkow et al., 2015; Muñiz et al., 2016).

### mPFC

Ample evidence indicates that the mPFC has a significant role in the development and persistence of addictive behavior (Volkow et al., 2012). By integrating input from cortical and subcortical areas and conveying excitatory output to the NAc, the mPFC is thought to exert control over the motor circuitry to regulate drug responses and drug-associated stimuli (Cadet and Bisagno, 2013). In preclinical models, mPFC is associated with reward, including cocaine-CPP, (Tzschentke and Schmidt, 1999) and salience- and novelty-detection (Dalley et al., 2004).

In the present study, we found that a combination of psychostimulants influenced the outcome of CPP. Molecular results from this study suggest that caffeine might strengthen environment-drug association within the mPFC-NAc network. Indeed, we found a pattern of IEGs mRNA expression similar to the one obtained from NAc tissue, i.e., increased mRNA of *cFos* and *Egr1* in the mPFC in the group that received the combination of both psychostimulants during CPP training. *cFos* levels are reported to be increased in mPFC neurons following exposure to a cocaine-associated environment (Slaker et al., 2015). In addition, cocaine-conditioned stimuli were associated with increased *Egr1* expression in the PFC (El Rawas et al., 2012).

In addition, we observed a cocaine-mediated decrease in *Drd2* mRNA following CPP and after chronic treatment in the home cage (in Experiment #3, all psychostimulants reduced *Drd2* expression in the mPFC). This finding is probably linked to chronic cocaine effects, in agreement with previous studies demonstrating a decrease in D2 receptor function in the mPFC is related to cocaine-induced sensitization (Beyer and Steketee, 2002). However, following acute psychostimulant administration, on the first conditioning day, we only found caffeine-mediated effects on *Drd1a* and *Drd2*. Both groups receiving caffeine, but not the group receiving cocaine alone, showed increased mRNA of these receptors in the mPFC. The differences in gene expression patterns seen in this study following acute and chronic drug administration suggest that specific neuroadaptations occurred in between those time points.

Among the differentially expressed IEGs seen in the mPFC compared to the NAc is *Npas4*. This IEG is expressed throughout the brain at a low level, though it is enriched in the frontal, parietal, and entorhinal cortices (Maya-Vetencourt et al., 2012; Spiegel et al., 2014). In our study, *Npas4* was only increased in the group that was injected with both psychostimulants during CPP training and following a single pairing CPP session, suggesting increases in mPFC activation.

Specific roles for *Npas4* in cocaine-mediated adaptations were reported by Ye et al. (2016). These authors demonstrated that there are brain-wide projection patterns separating mPFC cells responding to positive and negative experiences and that there was enrichment of *Npas4* expression in mPFC cells responding to cocaine. These authors also demonstrated that cocaine-induced increase of *Npas4* expression in mPFC is very rapid, within 30 min (Ye et al., 2016). It was proposed that *Npas4*, by controlling distinct networks of genes, differentially regulated synaptic input to excitatory and inhibitory neurons in order to facilitate circuit responses to sensory experience (Spiegel et al., 2014). *C*ognitive impairments are associated with decreased *Npas4* mRNA expression in the mouse frontal cortex (Qiu et al., 2016). Our results showing an association of CPP learning and increases in *Npas4* mRNA levels complement the decreases observed in cognitive decline.

We provide evidence here that caffeine may affect neuroplasticity through IEGs expression in circuits that are activated by drug use and associative learning. Our results also show that IEGs changes are specific to environmental context and that they are brain-region dependent. It is important for us to refocus from using these genes as activity markers, as has been done in the past, to exploring the role of these gene in establishing memory in mPFC cortical and cortical-subcortical circuits. Whilst this study describes specific changes in some early genes previously linked to memory and reward, it is difficult to establish the specific contribution of each of these genes to learning and memory since they may reflect general plasticity changes within the mPFC/NAc circuit.

Finally, it has been suggested that prevention of relapse might be achieved by using treatments that diminish the impact of drug-associated stimuli on drug seeking. Addiction is a brain disease and a learning disorder suggesting that drug-induced alterations can be undone or counteracted by a different learning strategy (Hyman et al., 2006). Interestingly, it has been reported that social interaction can prevent cocaine CPP (Prast et al., 2014).

This study also highlights the importance of considering caffeine effects as a potent adulterant in cocaine seized samples. Prieto et al. (2016) previously suggested that caffeine can act as a priming element for cocaine-related reward circuitry and as an effective discriminative stimulus for maintaining drug-seeking behavior in animals. In addition, caffeine intake is positively correlated with substance-use disorders (Kendler et al., 2006) and has been shown to increase illicit drug use (Miller, 2008). Thus, it seems reasonable to examine caffeine daily intake in psychostimulant-addicted patients undergoing treatment.

## Author contributions

VB, FU, CS, and BG were responsible for the study design. JM, JP, MS, and BG performed the experiments and analyzed data. VB, FU, BG, JLC, and CS provided critical revision of the manuscript for important intellectual content. All authors have critically reviewed content and approved final version submitted for publication.

### Conflict of interest statement

The authors declare that the research was conducted in the absence of any commercial or financial relationships that could be construed as a potential conflict of interest.
